# Accuracy and precision of responses to visual analog scales: Inter- and intra-individual variability

**DOI:** 10.3758/s13428-022-02021-0

**Published:** 2022-11-17

**Authors:** Miguel A. García-Pérez, Rocío Alcalá-Quintana

**Affiliations:** Departamento de Metodología, Facultad de Psicología, Universidad Complutense, Campus de Somosaguas, 28223 Madrid, Spain

**Keywords:** Likert scales, Visual analog scales, Pseudoneglect, Accuracy, Precision

## Abstract

Visual analog scales (VASs) are gaining popularity for collecting responses in computer administration of psychometric tests and surveys. The VAS format consists of a line marked at its endpoints with the minimum and maximum positions that it covers for respondents to place a mark at their selected location. Creating the line with intermediate marks along its length was discouraged, but no empirical evidence has ever been produced to show that their absence does any good. We report a study that asked respondents to place marks at pre-selected locations on a 100-unit VAS line, first when it only had numerical labels (0 and 100) at its endpoints and then when intermediate locations (from 0 to 100 in steps of 20) were also labeled. The results show that settings are more accurate and more precise when the VAS line has intermediate tick marks: The average absolute error decreased from 3.02 units without intermediate marks to 0.82 units with them. Provision of intermediate tick marks also reduced substantially inter- and intra-individual variability in accuracy and precision: The standard deviation of absolute error decreased from 0.87 units without tick marks to 0.25 units with them and the standard deviation of signed distance to target decreased from 1.16 units without tick marks to 0.24 units with them. These results prompt the recommendation that the design of VASs includes intermediate tick marks along the length of the line.

**Supplementary Information:**

The online version contains supplementary material available at 10.3758/s13428-022-02021-0.

Computer administration of questionnaires and inventories allows practicable replacement of Likert scales (LSs) with visual analog scales (VASs) so that respondents can indicate their position along a continuum rather than simply making a choice among a few discrete locations. Consider an item consisting of a statement about which respondents have to indicate their level of agreement. With a seven-point LS, a respondent would mark one of seven response options labeled from, say, −3 (or complete disagreement) to 3 (or complete agreement) in integer steps; in contrast, a VAS displays a line segment covering the same numerical range for the respondent to place a mark anywhere along the entire length of the line, limited only by the spatial resolution of the display. Then, in principle, the VAS allows respondents to express level of agreement with higher resolution, unlimited by the straitjacket of discrete integer locations. Indeed, Hayes, and Patterson (1921, p. 99) advocated the VAS (then referred to as the graphic rating method) because “the rator can make as fine a discrimination of merit as he chooses”, Ohnhaus and Adler (1975, p. 383) claimed that the VAS “reflects more precisely what a patient actually feels than the [Likert scale]”, Imbault, Shore, and Kuperman (2018, p. 2400) stated that the VAS “allows researchers to capture subtle individual differences that are lost in a [Likert scale]”, and Thomas, Manning and Saccone (2019, p. 5) declared that they used a VAS “in order to detect smaller differences in ratings compared to a traditional seven-point Likert scale.”

Replacing the discrete response set allowed by the LS with a quasi-continuous response set in the VAS is intuitively appealing, but claims that the VAS provides extra accuracy rest heavily on two implicit assumptions. One is that the respondent has a precise quantitative notion of what his/her exact position is anywhere in between the discrete landmarks provided by the LS, which are generally accompanied by verbal descriptions (e.g., mild disagreement, strong agreement, etc.). The second assumption is that the respondent is capable of identifying and marking the location along the line segment pertaining to that quantitative position.

The first assumption is impossible to test empirically for lack of a true measure of the presumed quantitative position held by the respondent that could be compared with the position that he/she reports. In addition, the assumption itself embodies the controversial notion that such a quantitative position actually exists (for a discussion of this notion, see Franz, 2022). Naturally, the research reported in this paper does not address the empirical validity of this assumption and focuses instead on the validity of the second one, which implies that a respondent intending to report, e.g., position 3.73 actually marks that exact location along a line whose left and right endpoints are labeled, e.g., 0 and 10, respectively. This assumption can be easily tested by checking the accuracy with which respondents mark the positions on the line that pertain to a set of numerical values given to them. Note that testing this assumption does not require respondents to come up with numerical values as subjective ratings of stimuli or materials submitted to their judgment. Instead, it only assesses respondents’ ability to mark given numerical values accurately on a VAS, which is a necessary condition to support claims of higher precision or better discriminability in the continuous VAS compared to the discrete LS.

There is a relatively large body of indirect and direct evidence pertaining to the empirical validity of this assumption. The largest set of data comes from studies of what Bowers and Heilman (1980) dubbed “pseudoneglect”, a characteristic by which neurologically normal subjects generally make left-sided errors when asked to mark the midpoint of a line. There is an overwhelming and diverse amount of evidence of pseudoneglect in visual line bisection (for reviews, see Friedrich, Hunter, & Elias, 2018; Jewell & McCourt, 2000; Kaul, Papadatou-Pastou, & Learmonth, 2021; Learmonth & Papadatou-Pastou, 2022) and the number of variables that moderate its magnitude is very large, including psychiatric conditions, action video gaming experience, or procedural characteristics of the visual bisection task (see, e.g., Bediou, Adams, Mayer, Tipton, Green, & Bavelier, 2018; Ciricugno, Bartlett, Gwinn, Carragher, & Nicholls, 2021; García-Pérez & Peli, 2014; Latham, Patston, & Tippett, 2014; Ochando & Zago, 2018; Rao, Arasappa, Reddy, Venkatasubramanian, & Reddy, 2015; Ribolsi, Di Lorenzo, Lisi, Niolu, & Siracusano, 2015; Saj, Heiz, Van Calster, & Barisnikov, 2020). We consider all of these data as indirect evidence against the assumption because they only corroborate that respondents *intending* to mark the midpoint of a line err at doing it, but this body of research does not provide any indication as to whether similar errors occur (and in what direction) when intending to mark alternative positions on the line. Nevertheless, the implications are serious when it comes to interpreting a mark made near the midpoint of a VAS line: In practice, it will never be known whether the respondent actually intended to mark the midpoint and is simply displaying a behavioral pseudoneglect bias or, instead, he/she is veridically expressing a position that is near but not exactly at the midpoint of the continuum. The latter case would attest to the extra resolution that the VAS allows (in comparison to an LS where the respondent would be forced to choose the central response option in such a case) but the former would indicate that pseudoneglect masquerades as extra resolution available on the VAS. The psychometric interpretation of VAS scores would be in further jeopardy if pseudoneglect bias occurred along the entire length of the line.

Dixon and Bird (1981) reported results that address this issue more directly. Eight subjects were shown a set of 10-cm vertical line segments each pre-marked at a specific reference position: 1, 2, 3, 4.6, 5, 5.5, 6, 7.5, 8.2, and 9.5 cm from the top. For each of these ten reference positions and in a random order, subjects were asked to reproduce the location of the mark, each on a new, unmarked line segment of identical length and orientation. Each respondent repeated the reproduction task seven times. Thus, arguably, the visually perceived position of the reference might play the role of the subjective magnitude elicited by an item on a questionnaire, which respondents then translate into a suitable mark that they make on a VAS. Average errors of reproduction (over subjects and repetitions) varied from −0.19 cm to 0.35 cm across reference positions, with underproduction at upper locations (at or under 5.5 cm from the top) and overproduction at lower locations (6 cm from the top and beyond). Variability of errors (again, across the 8 × 7 = 56 settings made for each reference) was also relatively large, with standard deviations ranging between 0.089 and 0.321 cm across reference positions. Thus, accuracy and precision vary along the length of the line at the group level. Unfortunately, measures of intraindividual performance were not reported (i.e., the mean and standard deviation across the seven repetitions made by each subject at each reference position), precluding an assessment of individual differences in the accuracy and precision with which each subject made his/her marks at different locations along the line. Note that this is actually of the utmost importance in an assessment of the suitability of the VAS for psychometric testing, where individual performance matters most and overall group performance is largely unimportant (in sharp contrast to survey studies in which the opposite holds; see Funke & Reips, 2012). For application in psychometric testing, whether the group as a whole makes accurate settings on average is largely unimportant, particularly if such eventuality arises because some subjects’ performance is strongly biased in one direction while that of others is strongly biased in the opposite direction.

More recently and more to the point, the specific assumption that this paper is concerned with (i.e., that subjects can mark intended positions precisely on a VAS line) was involved in the study of Reips and Funke (2008), though only at the group level. They had six groups of subjects (whose sizes varied between 46 and 64 members) mark 13 different values on a line segment. The six groups make a 3 × 2 between-subjects design in which line length (50, 200, or 800 pixels) was one of the factors and form of delivery of target values (percentage or ratio) was the other. In the percentage condition, the 13 values of concern were indicated as 5%, 10%, 20%, 25%, 33%, 40%, 50%, 60%, 67%, 75%, 80%, 90%, and 95% of the length of the line; in the ratio condition, the same 13 values were instead expressed as 1/20, 1/10, 1/5, 1/4, 1/3, 2/5, 1/2, 3/5, 2/3, 3/4, 4/5, 9/10, and 19/20 of the length of the line. Each subject in each condition made two marks for each value, the second one in a consecutive run over the same (pseudorandom) sequence of values in reverse order. The declared goal of Reips and Funke was to gather evidence that VAS scores provide an interval scale, which drove their collection and analysis of data away from the question of concern in the present paper. In particular, they reported that marked values were very close to target values across the board, with only minor and often negligible differences across conditions defined by the two factors under study. Yet, like Dixon and Bird (1981), they focused on overall group performance and they could not look at intraindividual variability (in this case because subjects performed only two settings per target value), nor could they look at the magnitude of individual differences in accuracy and precision along the length of the line.

The study reported here is a replication and extension of Reips and Funke (2008), with a focus on the magnitude of intraindividual variability and interindividual differences in accuracy and precision of settings across the length of the line. The study thus lines up with the goals of an earlier study that focused on the assessment of variability in bisection performance (Manning, Halligan, & Marshall, 1990). The replication part of the study used a VAS line unmarked except at the extremes, as in the original study of Reips and Funke. The extension involved the use of a VAS line with intermediate positions also marked on it. The main goal of this extension was to find out whether settings are comparatively more accurate with the help of these aids. Although, in principle, there is no strong reason to avoid the use of intermediate tick marks in practical administration of VASs, the use of unmarked lines was recommended from the very beginning. In fact, Freyd (1923, p. 99) listed a number of construction rules allegedly “based on experience” one of which was that “there should be no breaks or divisions in the line”, although no empirical evidence in support of this recommendation appears to have ever been provided (see, e.g., Scott & Huskisson, 1976). Maybe as a result of adherence to this unfounded recommendation, the unmarked VAS line has been used in virtually all studies (see, e.g., Bijur, Silver, & Gallagher, 2001; Downie, Leatham, Rhind, Wright, Branco, & Anderson, 1978; Flynn, van Schaik, & van Wersch, 2004; Funke & Reips, 2012; Guyatt, Townsend, Berman, & Keller, 1987; Hilbert, Küchenhoff, Sarubin, Nakawaga, & Bühner, 2016; Imbault, Shore, & Kuperman, 2018; Kuhlmann, Dantlgraber, & Reips, 2017; Lin, Manuel, McFatter, & Cech, 2016; Müssig, Kubiak, & Egloff, 2022; Warriner, Shore, Schmidt, Imbault, & Kuperman, 2017; Weigl & Forstner, 2021; Weigl, Schartmüller, Riener, & Steinhauser, 2021). Yet, in principle, the use of intermediate marks along the VAS line should not hamper performance; in fact, it makes sense that such lines can only improve performance by providing numerical anchor points along the scale.

The protocol of this study complies with the Declaration of Helsinki and obtained approval from the institutional ethics committee. Data and materials for this study are available at https://osf.io/96wtm.

## Method

### Subjects

Thirty-five subjects (16 males and 19 females) participated in the study, including the two authors (subjects #1 and #2, one of each sex). Except for the authors, all subjects were naïve to the goals of the study. Their ages ranged from 19 to 62 years with an average of 35.3 years and a standard deviation of 16.4 years. They all signed an informed consent form prior to participation. Data from the two authors are available in the OSF repository but they are not used here. Thus, the effective sample for all statistical analyses consists of 33 subjects.

### Materials

The VAS response line was displayed on a 28-inch, BenQ EL2870U LED monitor (screen size: 62.2 × 34.4 cm; spatial resolution: 3840 × 2160 pixels; frame rate: 60 Hz). matlab scripts that called Psychophysics Toolbox Version 3 (http://psychtoolbox.org) functions governed stimulus presentation and response collection during experimental sessions. The black (gray level 0) VAS line was 1001 pixels long (16.2 cm on the face of the monitor) and 11 pixels thick and it was displayed vertically and horizontally centered on the screen on a light gray background (gray level 200) that covered the entire image area. The left and right ends of the line were each 1420 pixels (23 cm) away from the corresponding edge of the image area. A VAS line 1001 pixels in length (which was unknown to respondents) was chosen for two reasons. One was to allow high-resolution measurement of the location marked by the respondent (not to be mistaken for high response resolution on their part); the second reason was to preclude respondents from using pixel-counting strategies to give the “correct” response on each trial. The labels “0” and “100” were displayed in black beneath the left and right edges of the VAS line, respectively, to serve as a reminder to the respondents of the numerical values assigned to those positions. The labels were 0.7 cm in height, their top part was 0.9 cm below the VAS line, and they were horizontally centered with the applicable edge of the VAS line. The target value whose location the respondent had to mark on each trial was displayed in text reading “Mark position XX” (but in Spanish), where XX was replaced with the corresponding numeral. This text string was horizontally centered with the left end of the VAS line and was displayed in green (RGB triplet: [51 153 51]). The height of the characters was 1 cm and the baseline of the text was located 8.5 cm above the VAS line.

The ten target values that respondents had to mark on the VAS line were 7, 16, 28, 37, 43, 55, 69, 72, 84, and 93 units from the left end of the line. The potential advantage of the VAS over an LS relies on the fact that subjective magnitude can be anywhere within the numerical response range and, thus, the values just listed are as plausible or intrinsically interesting as any others can be. Yet, these values avoid the landmarks conventionally implied in LSs (i.e., tenths, fifths, quarters, or thirds of the span) and, thus, they provide information on how capable respondents are of locating, say, 55 when they try to express a distinction by marking 55 and not 50. Note also that our set includes the values 69 and 72 so that the data will indicate the extent to which respondents can accurately report small differences. Target values were presented sequentially in a random order only constrained to ensure that no pair of consecutive trials involved successive numbers on the ordered list. Each respondent went through the set of target values ten times with discretional breaks between blocks and with the order of target values newly randomized for each respondent in each block.

### Procedure

Respondents sat straight in front of the display to maintain an approximate viewing distance of 70 cm so that the horizontal VAS line subtended about 13 degrees of visual angle. Their heads were not restrained but they were asked to refrain from changing viewing distance or angle by any meaningful amount throughout the session. To mimic the natural conditions of computer administration of psychometric tests or surveys with VAS items, data-collection sessions were conducted under standard office lighting with the precaution to prevent reflected glare on the display screen. For the same reason, respondents did not undergo practice trials with which they could calibrate or adjust their performance before data collection. They were nevertheless allowed to gain familiarity with the trial design and the response interface by providing three consecutive sets of three trials with target values 0, 50, and 100. These should not have allowed any actual calibration of their performance, but the responses to the target of 50 units provide data on bisection ability for an informal assessment of pseudoneglect.

Respondents signed an informed consent form after they had been briefed that the goal of the study was to investigate human perception of visual space through our ability to identify and mark relative locations along a straight line. They were then shown the layout of the line with the “0” and “100” labels placed as described above and they were told that on each trial they had to place the mouse cursor at the position on the line corresponding to the target value indicated at the top of the display. Display of the default mouse cursor was disabled and replaced with a vertical line segment centered on the VAS line. This “slider” was 61 pixels in vertical length, 3 pixels in horizontal width, red in color (RGB triplet: [255 0 0]), and it was positioned on the left end of the VAS line at the beginning of each trial. Mouse movements only affected the horizontal position of the slider along the VAS line and its horizontal range of movement was limited to the length of the VAS line. Respondents could move the mouse back and forth and they had to click on the left mouse button to enter their setting at the location they judged appropriate, which would then give way to the next trial for another target value. Confirmation of the setting was not required and there was no chance to alter the setting once made.

Past a short break after the session just described was completed, subjects went through a second session of identical characteristics except that the VAS line now had tick marks on it at positions ranging from 0 to 100 in steps of 20. The tick marks were thin, black vertical lines 3 pixels in horizontal width and vertically spanning from 15 pixels above the center of the VAS line to 15 pixels below it. Each tick mark was also labeled with the corresponding value (i.e., 0, 20, 40, 60, 80, and 100) in the same manner as labels “0” and “100” were displayed for the otherwise unmarked VAS line used in the first session. The session with the unmarked VAS line was always run first to avoid the effects that practice with a marked VAS line could have on subsequent performance with an unmarked line. No additional “familiarization” trials were allowed prior to the beginning of this second session.

### Data analysis

Ten subjects reported occasional and unintentional clicking of the left mouse button while moving it to make their setting. These eventualities could be identified and the stray settings were obvious candidates for removal, but we also looked for evidence of analogous errors that might not have been reported by our subjects. Data were first visually inspected by displaying them as shown in Supplementary Figs. S2 and S3, which revealed some settings that were unlike the remaining ones in each set of ten. In a study on variability across subjects, target locations, and conditions, it does not seem appropriate to remove settings based on the standard deviation of any overall distribution. Thus, candidates for removal had to be tagged separately within the set of ten settings for each subject, target location, and condition. We found out that using the off-the-shelf criterion of three standard deviations (SDs) away from the mean, no setting whatsoever was tagged. We determined that the criterion of 2.7 SDs away from the mean tagged settings that looked like reasonable outliers. Supplementary Fig. S1 lists all the settings that were finally removed, which included most of the cases declared by the subjects themselves as erred settings.

The concordance correlation coefficient ρ_*c*_ (see Lin, 1989; Lin, Hedayat, Sinha, & Yang, 2002) between all target values (*T*) and settings (*S*) was computed using data from each subject and condition separately. The concordance correlation coefficient measures agreement between variables via the spread of data around the identity line and it combines measures of accuracy and precision. The coefficient is defined as

$${\uprho}_c=\frac{2{r}_{st}{s}_s{s}_t}{s_s^2+{s}_t^2+{\left(\overline{S}-\overline{T}\right)}^2},$$

where the right-hand side uses standard symbols for means, standard deviations, variances, and correlation for the two variables of concern.

A separate measure of the precision of settings (i.e., their dispersion) was obtained for each subject at each target position and condition via the average absolute error (*AAE*) defined as $AA{E}_{ijc}=\frac{1}{n_{\textrm{S}}\kern0.1em }\sum\limits_{k=1}^{n_{\textrm{S}}}\left|{S}_{ijc k}-{T}_j\right|$, where *i* is the subject index, *j* is the target index (*j* = 1, …, 10), *c* is the condition index (*c* ∈ {U, M}, for unmarked and marked VAS lines), *k* is the setting index (*k* = 1, …, *n*_S_, where *n*_S_ is the number of valid settings made by subject *i* with target *j* in condition *c*, with 9 ≤ *n*_S_ ≤ 10), and *S*_*ijck*_ is the *k*-th setting made by subject *i* for target *j* in condition *c*. An overall measure of precision for each subject in each condition was subsequently defined as $AA{E}_{ic}=\frac{1}{n_{\textrm{T}}\kern0.1em }\sum\limits_{j=1}^{n_{\textrm{T}}} AA{E}_{ijc}$, where *n*_T_ = 10 is the number of target values.

A separate measure of the relative accuracy of settings (i.e., their overall distance to the corresponding target values) was also obtained for each subject at each target position and condition via the average distance *D* across settings, defined as ${D}_{ijc}=\frac{\sum\limits_{k=1}^{n_{\textrm{S}}}{S}_{ijc k}}{n_{\textrm{S}}\kern0.1em }-{T}_j$. An overall measure of accuracy for each subject in each condition was analogously defined as ${D}_{ic}=\frac{1}{n_{\textrm{T}}\kern0.1em }\sum\limits_{j=1}^{n_{\textrm{T}}}{D}_{ijc}$.

## Results

**Fig. 1:**
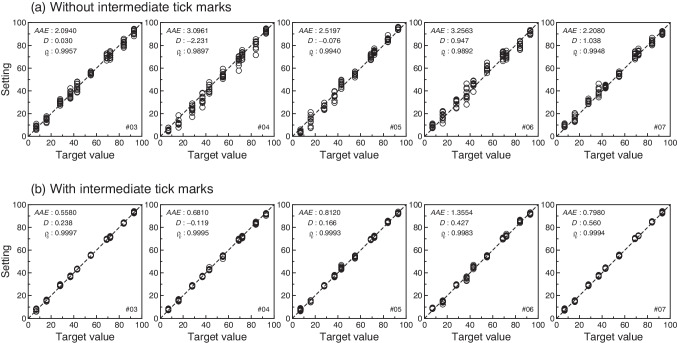
Scatter plot of settings against target values for five sample subjects in the conditions without tick marks (**a**) and with them (**b**). A diagonal identity line is plotted for reference. The set of *vertically aligned symbols* at each target value represent the settings made for the corresponding target across ten repetitions. The *inset* in each panel indicates the value of the overall index of precision (*AAE*), the overall index of accuracy (*D*), and the concordance correlation coefficient ρ_c_. Data for the remaining subjects are available in Supplementary Figs. S2 and S3

Figure 1 shows a scatter plot of the settings *S*_*ijck*_ made across target locations by the same five subjects with unmarked VAS lines (Fig. 1a) and marked VAS lines (Fig. 1b). Analogous plots for all subjects are provided in Supplementary Figs. S2 (unmarked VAS) and S3 (marked VAS). It is immediately obvious that settings are, for each and all subjects, much more tightly packed around the corresponding target values when the VAS line is marked along its length. A comparison of settings with and without marked VAS lines for our close targets of 69 and 72 units is also informative. With an unmarked line, some subjects (e.g., #3 and #6 in Fig. 1a; see also data for subjects #12, #14, and #17 in Supplementary Fig. S2) produce settings whose distributions display substantial overlap for these two target values and, in some cases, the two distributions are virtually identical (see subjects #6 and #14 in Supplementary Fig. S2); in contrast, the same subjects produce separate distributions of settings at each of these targets when the VAS line is marked (compare with data from the corresponding subjects in Fig. 1b and in Supplementary Fig. S3, where apparent overlap is only caused by the size of the symbols used to plot individual settings). Other subjects (e.g., #4 in Fig. 1a) produce less overlapping distributions of settings at these two target values with unmarked lines, but the distance between the distributions is expanded in comparison to the distance between targets; in contrast, with marked lines (see Fig. 1b and Supplementary Fig. S3), the settings provided by these subjects are more on target, just as they are for all other subjects.

**Fig. 2:**
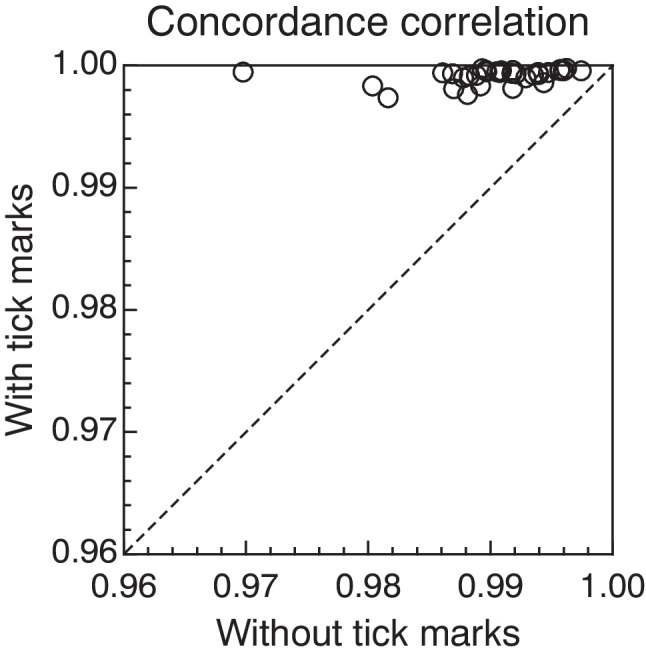
Scatter plot of concordance correlation between targets and settings for each subject in the conditions without intermediate tick marks (*horizontal axis*) and with them (*vertical axis*). The diagonal identity line is plotted for reference

Concordance correlation coefficients for each subject (see the inset in each panel of Fig. 1 and Supplementary Figs. S2 and S3) are generally very high whether with or without tick marks, mostly due to the broad spread of target values compared to the smaller variability of settings at each target value. Nevertheless, the variability of settings at any individual target value is visibly much smaller with tick marks than without them, something that transfers to the values of ρ_*c*_. Figure 2 plots the value of ρ_*c*_ with tick marks against the value of ρ_*c*_ without tick marks across subjects, revealing that the agreement between settings and targets is invariably larger in the former condition (i.e., all data points lie above the diagonal identity line). It is thus immediately obvious that the difference between the concordance correlation with tick marks and that without them is positive for each and all of the subjects. It is well known that in these conditions any statistical test of the null hypothesis that differences are zero will be rejected at any reasonable alpha level but, at the request of a reviewer, we conducted a paired-samples *t* test of equality of means of concordance correlations with the predictable significant outcome at α = .05 (*t*_32_ = 9.55; *p* < 10^−10^; CI_95_: [0.007, 0.011]). The effect size was also large (Cohen’s *d*_*z*_ = 1.66).

**Fig. 3:**
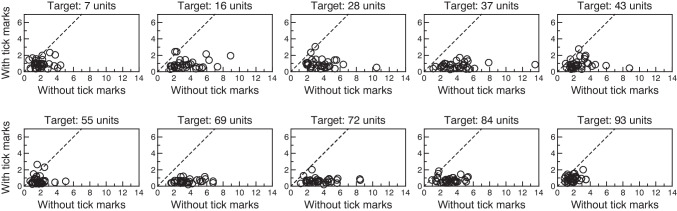
Scatter plot of precision with intermediate tick marks (average absolute error *AAE*_*ij*M_) against precision without them (average absolute error *AAE*_*ij*U_) at each target position (*panels*). Each symbol in each panel comes from a different subject. Note that the vertical axis is half the size of the horizontal axis in each panel in order to accommodate differences in ranges without distorting the scales across axes. The 45-deg identity line is plotted for reference

Figure 3 compares precision with and without tick marks by plotting average absolute error with tick marks (*AAE*_*ij*M_) against average absolute error without them (*AAE*_*ij*U_) for all subjects at each target location. With rare exceptions, *AAE*_*ij*M_ is meaningfully smaller than *AAE*_*ij*U_ for all subjects and target locations (i.e., most data points are located below the 45-deg identity line in each panel). The improvement in precision with tick marks is minimally smaller for targets located near either end of the VAS line (locations 7 and 93). Across the board, *AAE*_*ij*U_ ranged from 0.58 to 13.54 units whereas *AAE*_*ij*M_ ranged instead from 0.20 to 3.04 units. We also conducted here paired-samples *t* tests for means comparing average absolute errors with and without tick marks separately at each target location. All tests came out significant at α = .05. Test statistics ranged from *t*_32_ = 6.01 (at target location 7; leftmost panel in the top row of Fig. 3) to *t*_32_ = 12.91 (at target location 69; second panel from the left in the bottom row of Fig. 3), all *p* values were lower than 10^−5^, and effect sizes varied from *d*_*z*_ = 1.05 (at target location 7) to *d*_*z*_ = 2.25 (at target location 69). Note that all tests would also have been significant if we had used a thoroughly inappropriate correction for multiple testing that sets the threshold *p* value at α/10 = .005. An overall picture of precision collapsed across target locations is presented below in Fig. 5a.

**Fig. 4:**
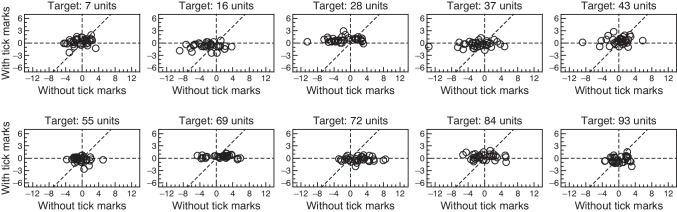
Scatter plot of accuracy with intermediate tick marks (average distance to target *D*_*ij*M_) against accuracy without them (average distance to target *D*_*ij*U_) at each target position (*panels*). Each symbol comes from a different subject. Note that the vertical axis is half the size of the horizontal axis in each panel in order to accommodate differences in ranges without distorting the scales across axes. *Cross lines* through the origin and a 45-deg identity line are plotted for reference

**Fig. 5:**
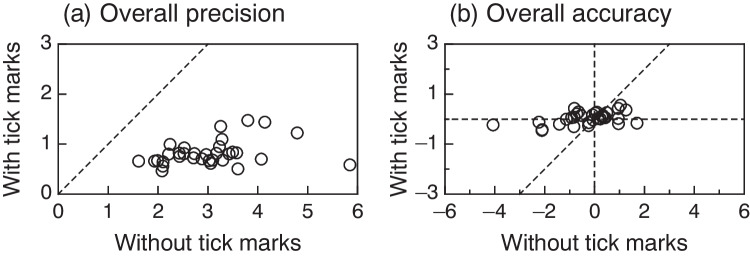
Scatter plot of overall precision (**a**) and overall accuracy (**b**) with and without tick marks. Each symbol represents a different subject. Note that the vertical axis is half the size of the horizontal axis in each panel in order to accommodate differences in ranges without distorting the scales across axes. The 45-deg identity line in each panel and the cross lines at the origin in the right panel are plotted for reference. Symbol position along the horizontal dimension represents the overall measure of average absolute error (*AAE*_*i*U_) in the left panel and the overall measure of distance to target (*D*_*i*U_) in the right panel for settings made without tick marks. Symbol position along the vertical dimension represents analogous indices of accuracy and precision (*AAE*_*i*M_ and *D*_*i*M_) for settings made with tick marks

Figure 4 compares accuracy with and without tick marks by plotting average distance to target with tick marks (*D*_*ij*M_) against average distance to target without them (*D*_*ij*U_) for all subjects at each target location. Again, *D*_*ij*M_ is more tightly packed around 0 than *D*_*ij*U_ is. Across the board, *D*_*ij*U_ ranged from −13.54 to 8.47 units whereas *D*_*ij*M_ spanned the much narrower range from −2.64 to 2.94 units. Only for the target located at 93 units are *D*_*ij*M_ and *D*_*ij*U_ similarly distributed around zero, perhaps due to the anchoring reference provided by the label “100” displayed immediately below the right end of the VAS line and horizontally centered with it. Although the horizontal spread of data points in each panel of Fig. 4 is meaningfully larger by eye than its vertical spread, we conducted Pitman–Morgan tests of equality of variances with and without tick marks at each target location. At α = .05, all tests came out significant with test statistics ranging from *t*_31_ = 5.53 (at target location 93) to *t*_31_ = 21.48 (at target location 69) and with all *p* values lower than 10^−5^. An overall picture of accuracy collapsed across target locations is presented below in Fig. 5b.

For an overall comparison of precision with and without tick marks, Fig. 5a plots average absolute error with tick marks aggregated over target locations (*AAE*_*i*M_) against average absolute error without tick marks aggregated over target locations (*AAE*_*i*U_) for each subject. Quite apparently, overall precision with tick marks greatly exceeds precision without them: In Fig. 5a, *AAE*_*i*M_ ranges from 0.46 to 1.48 units with an average of 0.82 units and a standard deviation of 0.25 units; in contrast, *AAE*_*i*U_ ranges from 1.61 to 5.85 units with an average of 3.02 units and a standard deviation of 0.87 units. In addition, each and all subjects exhibit more precision with tick marks than without them: All data points are located below the 45-deg identity line. Although Fig. 5a speaks for itself in this respect, a paired-samples *t* test for means comparing overall precision with and without tick marks unsurprisingly revealed statistically significant differences (*t*_32_ = −15.15; *p* < 10^−15^; CI_95_: [−2.49, −1.90]) and a large effect size (Cohen’s *d*_*z*_ = 2.64). Also, in accordance with the observable differences in vertical and horizontal scatter of data in Fig. 5a, the variance of precision with and without tick marks differed significantly by the Pitman–Morgan test (*t*_31_ = −9.57; *p* < 10^−10^).

For an analogous overall comparison of accuracy with and without tick marks, Fig. 5b plots average distance to target with tick marks aggregated over target locations (*D*_*i*M_) against average distance to target without tick marks aggregated over target locations (*D*_*i*U_) for each subject. In Fig. 5b, *D*_*i*M_ ranges from −0.45 to 0.56 units with an average of 0.05 units and a standard deviation of 0.24 units whereas *D*_*i*U_ ranges from −4.07 to 1.70 units with an average of −0.28 units and a standard deviation of 1.16 units. One-sample *t* tests for means revealed that, at α = .05, the average *D*_*i*M_ does not differ significantly from zero (*t*_32_ = −1.38; *p* = 0.177; CI_95_: [−0.70, 0.13]), nor does the average *D*_*i*U_ (*t*_32_ = 1.11; *p* = 0.274; CI_95_: [−0.04, 0.13]). A paired-samples *t* test for means comparing overall accuracy with and without tick marks revealed that the difference is not statistically significant either (*t*_32_ = 1.76; *p* = 0.088; CI_95_: [−0.05, 0.71]). Essentially, these three results imply that overall bias is similar and nearly absent whether with or without tick marks. On the other hand, and in agreement with what Fig. 5b shows, the variance of accuracy with and without tick marks differed significantly by the Pitman–Morgan test (*t*_31_ = −14.82; *p* < 10^−14^).

**Fig. 6:**
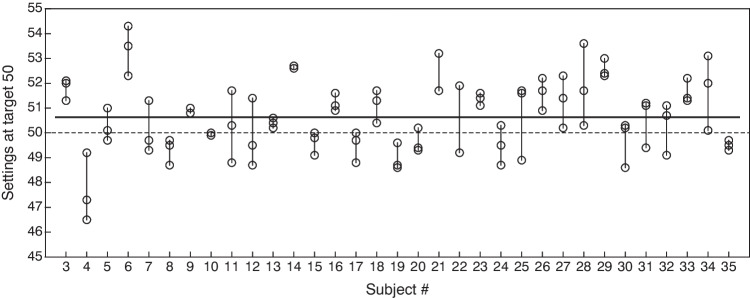
Settings (*symbols*) at target 50 (*midpoint*) in the unmarked VAS line for each subject. The *horizontal dashed line* across the panel indicates the target value; the *solid line* at an ordinate of 50.63 units indicates the average value of all settings (the median was 50.5)

The fact that accuracy without tick marks, both at each individual target (Fig. 4) and overall (Fig. 5b), averages around zero across subjects suggests that there are no major spatial distortions in the perceived location of each target at the group level. This result has some bearing on the issue of pseudoneglect that was discussed in the introduction, or its generalization to locations other than the midpoint of a line. We thus looked for evidence of pseudoneglect (as originally defined) in the data collected during the practice phase, which requested three settings for a target value of 50 units (the midpoint) with an unmarked line. Figure 6 shows the settings made by each of the subjects for this target. There are obvious individual differences in variability across repeated settings (compare, e.g., the variable settings made by subjects #4 and #11 with the almost invariant settings made by subjects #13 and #35). There are also individual differences in that some subjects place their settings on one or the other side of the true target (compare, e.g., subjects #4 and #6). Overall, however, there is no sign of any meaningful form of pseudoneglect at the group level: The average setting across subjects was 50.63 units (the median was 50.5 units), only minimally to the right of the true midpoint and certainly not to the left (95% CI: [50.34, 50.92]). Whether or not this result is generalizable is unclear, particularly in the light of the diversity of results reported across studies on pseudoneglect (see Friedrich et al., 2018; Jewell & McCourt, 2000; Learmonth & Papadatou-Pastou, 2022). In any case, the presence of spatial biases in some form and magnitude cannot be ruled out at the individual level, which is the relevant unit of analysis when VASs are used to collect psychometric data.

We also looked for similar patterns of directional misplacement of settings at each of the non-central locations in our set of ten targets. In fact, a cursory look across the panels of Supplementary Fig. S2 (for settings without intermediate tick marks) shows that some subjects display clear signs of leftward bias at locations below the midpoint, with or without signs of the opposite directional bias at locations above the midpoint (e.g., subjects #25, #31, or #35). Other subjects do not show differences in directional bias at locations below and above the midpoint (e.g., subjects #5 or #10). No such patterns are immediately obvious in the panels of Supplementary Fig. S3 (for settings with tick marks), because settings in these conditions are much closer to target.

**Fig. 7:**
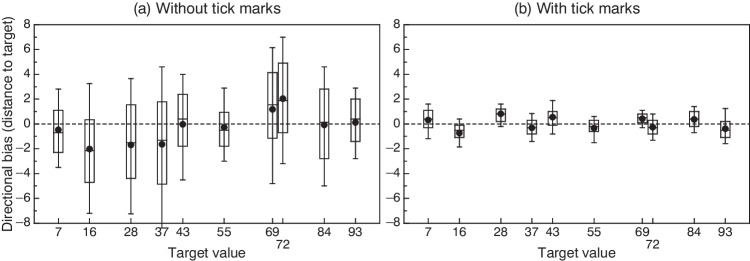
Box plots for overall distributions of settings at each target location with the unmarked VAS line (**a**) or the marked VAS line (**b**), expressed as distance to the corresponding target. Each box plot summarizes a maximum of 330 settings (33 subjects × 10 repetitions) and a minimum of 324 settings in cases in which outlying settings had been removed. *Negative values along the vertical axis* represent leftward bias; *positive values* represent rightward bias. The *bottom* and *top edges* of each box indicate the 25th and 75th percentiles, the *central horizontal line* indicates the median, and the *whiskers* extend from the 10th to the 90th percentiles. A *circle* indicates the mean

Although individual differences matter when responses to psychometric tests are collected with VAS items, we checked informally whether our data at the group level show a pattern of directional bias similar to that reported by Dixon and Bird (1981) and discussed in the Introduction: Using a vertically oriented line with the origin at the top, they reported underproduction at upper locations and overproduction at lower locations. If this pattern is related to the numerical scale of the line regardless of its orientation, it would translate into leftward bias below the midpoint and rightward bias above the midpoint of our horizontal line. Figure 7 shows box plots of settings at each target location with data aggregated across subjects and repetitions in each condition regarding intermediate tick marks. In Fig. 7a, evidence of overall leftward bias (alternatively, rightward bias) is clearly apparent at intermediate locations within the left side (alternatively, right side) of the VAS line. Directional bias is less apparent and certainly weaker near either end of the VAS line (target locations 7, 84, and 93) or near its midpoint (target locations 43 and 55). With marked lines (Fig. 7b), bias is negligible (less than ± 0.9 units at all target locations) and the placement of the distributions minimally on the left or the right of target does not display any pattern. Although no hypotheses motivate this descriptive analysis, we report for the record that bias did not differ significantly (α = .05) from zero for targets 43, 84, and 93 in the condition without tick marks; in most other cases, *p* values were lower than 10^−5^. As regards pairwise comparisons in the condition without tick marks (Fig. 7a), locations at which strong leftward bias was apparent (targets 16, 28, and 37) did not differ significantly (α = .05) from one another in average bias but each of them differed from all the rest (all *p* values lower than 10^−4^). Analogously, locations at which rightward bias seemed to occur (targets 69 and 72) differed significantly in average bias from one another (*p* < .001) and each of them also differed from all the rest (all *p* values lower than .001). The only other significant comparisons involved locations 7 and 43 (*p* = .042), locations 7 and 93 (*p* = .013), and locations 55 and 93 (*p* = .038). In the condition with tick marks, where bias is negligible across the board (see Fig. 7b), the tight distributions resulted in significant differences in about half of the pairwise comparisons but this outcome is not empirically relevant.

## Discussion

Our study investigated interindividual and intraindividual variability in the accuracy and precision of settings corresponding to target numerical positions on a line marked only at the extremes or also with intermediate marks along its length. As anticipated, overall accuracy and precision increased and both types of variability were substantially reduced when settings were aided by the presence of intermediate tick marks. On the other hand, such improvements in performance did not vary across target positions along the length of the line. In quantitative terms, overall precision (average absolute error) on a 100-unit line without tick marks ranged across subjects from 1.61 to 5.85 units (mean, 3.02 units; SD, 0.87 units) whereas the addition of intermediate marks increased precision by reducing average absolute error down to a range between 0.46 and 1.48 units (mean, 0.82 units; SD, 0.25 units). On the other hand, overall accuracy (signed average distance to target) on the 100-unit line without tick marks ranged across subjects from –4.07 to 1.70 units (mean, –0.28 units; SD, 1.16 units) whereas the addition of intermediate marks increased accuracy by reducing signed average distance down to a range between –0.45 and 0.56 units (mean, 0.05 units; SD, 0.24 units). These results have immediate implications for the design of VAS items in psychometric testing.

The accuracy and precision with which respondents make a setting at the location where they intend to make it is higher if intermediate marks are provided along the length of the VAS line. It is noteworthy that use of VAS items in psychometric testing has not included intermediate marks (see, e.g., Simms, Zelazny, Williams, & Bernstein, 2019; Toland, Li, Kodet, & Reese, 2021), perhaps because use of such marks was explicitly and unfoundedly discouraged from the very beginning (Freyd, 1923) and subsequently. Thus, on describing the use of VAS in health research, McDowell (2006, p. 580) emphasized that “to produce a smooth response distribution, the VAS generally does not include numbers along the scale. This is because people often favor numbers ending in zero or five, which produces a stepped distribution of responses.” (This response style is referred to as “heaping”; see Furukawa, Hojo, Sakamoto, & Takaoka, 2021.) Furthermore, on describing their use of a VAS response format, Lin et al. (2016, p. 50) emphasized that “no numbers were visible to participants. This was to prevent participants from paying attention to the numbers so that they could focus primarily on their perception of changes in response.” Although these comments are intuitively appealing, no evidence seems to have ever been reported in their support. A second reason for the omission of intermediate marks may lie in that virtually all research conducted on the accuracy of VAS responses has systematically avoided them (see references to this effect in the Introduction). In contrast, our results indicate that the presence of intermediate marks along the VAS line brings meaningful increases in precision and accuracy of settings as well as a reduction of intraindividual and interindividual variability in these respects. It is thus noteworthy that this modification of the response format produces improvements of much more substance than those that have been sought for via other alterations of the response format with unmarked VAS lines (see, e.g., Maineri, Bison, & Luijkx, 2021; Reips & Funke, 2008; Revill, Robinson, Rosen, & Hogg, 1976; Scott & Huskisson, 1976, 1979).

It should be emphasized that our study does not rest on the assumption that respondents’ opinions, levels of agreement, etc. with respect to the content of a questionnaire item exist in the form of numerical magnitudes, nor did our study attempt to elucidate whether this is the case. We only aimed at investigating whether the conventional interpretation of VAS scores as numerical indicators of quantitative characteristics is supported by empirical evidence of respondents’ ability to mark intended numerical positions on a VAS line. A researcher’s ability to measure with exquisite precision the location of marks made on a line segment should never be misconstrued as a precise expression of the respondents’ numerical translation of their subjective ratings. Yet, in practice, the location of marks made on a VAS line are always physically measured with precision and interpreted as reflecting the actual magnitude that subjects intended to indicate, although sometimes data are subsequently polychotomized for convenience (e.g., Flynn et al., 2004; Toland et al., 2021; Hyland, Shevlin, McBride, Murphy, Karatzias, Bentall, Martinez, & Vallières, 2020; van Laerhoven et al., 2004).

Regarding the presumed accuracy that accompanies VAS responding, Simms et al. (2019) expressed skepticism with the widespread belief that “humans can make fine-grained distinctions along [visual analog] scales in a way that actually improves precision” (p. 559). They analyzed the psychometric properties of questionnaires alternatively consisting of VAS items or Likert-type items with different numbers of response options and the results of their study reportedly “failed to show any psychometric advantage for visual analog items relative to traditional Likert-type items” (p. 564). This led them to conclude that “the promise of added psychometric precision is not realized in practice with scales based on visual analog items, perhaps because humans are unable to reliably make meaningful and valid fine-grained distinctions for coarse items reflecting complex psychological characteristics” (p. 565). It is nevertheless fair to say that their results did not advise against the use of VASs; the results only failed to show any beneficial effect on the resultant reliability or validity of personality inventories. In addition, their study used unmarked VAS lines; a still open question is whether the extra accuracy and precision of settings in the presence of intermediate marks (as shown here for numerical targets) improves the properties of psychometric scales when respondents express instead subjective ratings.

On another front, VAS items lend themselves to item response theory (IRT) analysis under the continuous response model (Mellenbergh, 1994; Müller, 1987; Samejima, 1973) and freeware for parameter estimation under this model is available (Zopluoglu, 2012). We are only aware of one paper reporting the use of this IRT model for VAS responses (Liu, Peterson, Wing, Crump, Younger, Penner, Veljkovic, Foggin, & Sutherland, 2019), but their study did not include a comparison with responses to traditional Likert-type items. Although both LSs and VASs rest on the contentious assumption of an underlying quantitative psychological continuum that respondents have access to, it remains to be seen whether discrete (e.g., the graded response model) or continuous response IRT models offer similar characterizations of the psychometric properties of psychological tests whose items are administered in either Likert or VAS formats.

It should be noted that we have referred to a slider as the method by which subjects make their setting on a VAS line. Sliders and VASs have sometimes been referred to as alternative methods, with the distinction mostly reflecting arbitrary decisions regarding the action needed to give a response in each case (i.e., drag-and-drop versus move-and-click; see Table 1 in Funke, 2016). In this respect, the aspects that presumably create problems with sliders were not included in the design of our interface, with which the response format was identical to that involved in the typical VAS format (i.e., move the mouse cursor to the desired location and click to enter the setting). We have no reason to think that this choice of response format may have had any influence in our results and, specifically, on the substantial improvement in accuracy and precision that we have shown to accompany the provision of intermediate tick marks along the length of the VAS line.

Our study involved an arbitrarily defined set of ten targets, whose values were selected with the only criterion to avoid easily identifiable landmarks such as tenths, quarters, etc., of the length of the line. We do not see in the specific values that we selected any distinctive feature that could have affected our results in a way that would not have occurred with other choice of target values that also avoid landmarks. Thus, the reported increase in accuracy and precision of settings as well as the reduction of interindividual and intraindividual variabilities that come with the inclusion of intermediate tick marks are unlikely to be differentially related to the particular set of target values selected.

We also see no reason that these results would vary meaningfully if the number of intermediate tick marks were larger (e.g., every ten units instead of every 20 units along the length of the VAS line). If anything, one would certainly expect accuracy and precision to be even higher (and intraindividual and interindividual variabilities to be lower) by provision of further intermediate marks. The most extreme case in this respect implies the provision of numerical feedback as to the exact location of the slider as it moves (see, e.g., Figure 3 in Couper, Tourangeau, Conrad, & Singer, 2006). In these circumstances, subjects asked to mark position, say, 37 will certainly and invariably enter their setting when feedback indicates that the slider is at that precise position. Naturally, the question of interest is whether a larger number of intermediate marks (or the provision of positional feedback) will help respondents mark the position they intend when they are asked to indicate a subjective magnitude and not just to reproduce a numerical location given to them. This, again, bears on the issue of whether such a subjective magnitude actually exists and is available to the respondent. This may not be the case. In fact, studies in which feedback was or was not provided to aid respondents when expressing subjective magnitudes on a VAS line have shown that respondents receiving feedback selected round numbers meaningfully more frequently than respondents who did not receive feedback (Couper et al., 2006; Maineri et al., 2021). All things considered, creating VAS lines that include a relatively small number of intermediate marks (i.e., every 20 units for a scale ranging from 0 to 100) seems a reasonable compromise between aiding respondents to place their intended marks with precision and simultaneously preventing them from stereotypically selecting positions that are multiples of five or ten units.

### Supplementary information

ESM 1 (PDF 115 kb)
