# Supplementary material for: Accuracy and precision of responses to visual analog scales: Inter- and intra-individual variability
Source: Behav Res Methods. 2022 Nov 17;55(8):4369–81. doi: 10.3758/s13428-022-02021-0 (PMC10700476; doi:10.3758/s13428-022-02021-0)
Supplement: Supplementary file 1 — (PDF 115 kb) [file 13428_2022_2021_MOESM1_ESM.pdf]

Accuracy and precision of responses to visual analog scales: Inter- and intra-individual variability

Supplementary figures

Behavior Research Methods
https://doi.org/10.3758/s13428-022-02021-0

Miguel A. García-Pérez and Rocío Alcalá-Quintana
Departamento de Metodología, Facultad de Psicología, Universidad Complutense,
Campus de Somosaguas, 28223 Madrid, Spain

Removed settings in unmarked VAS
Removed settings in marked VAS

Target value

Subject	7	16	28	37	43	55	69	72	84	93
4				0.0						
5			0.0							
6				7.7				92.7		0.0
8		11.4	37.8					77.6		
10					50.7					
12	8.1	0.0				51.6			88.6	
13									89.2	
14		11.4	11.1	23.2						
17			37.3		45.3					
19		10.4						72.8		
20	11.3	11.1							89.4	
21					40.1					
22			27.6		0.0				76.9	
23								64.6 91.4		
28		10.8							56.6 89.7	
31				26.8						
32								0.0		
35									87.5	

Supplementary Figure S1. Settings (numerals in cells) removed for some subjects (rows) at some target values (columns) in the condition without intermediate tick marks (red) and with them (blue).

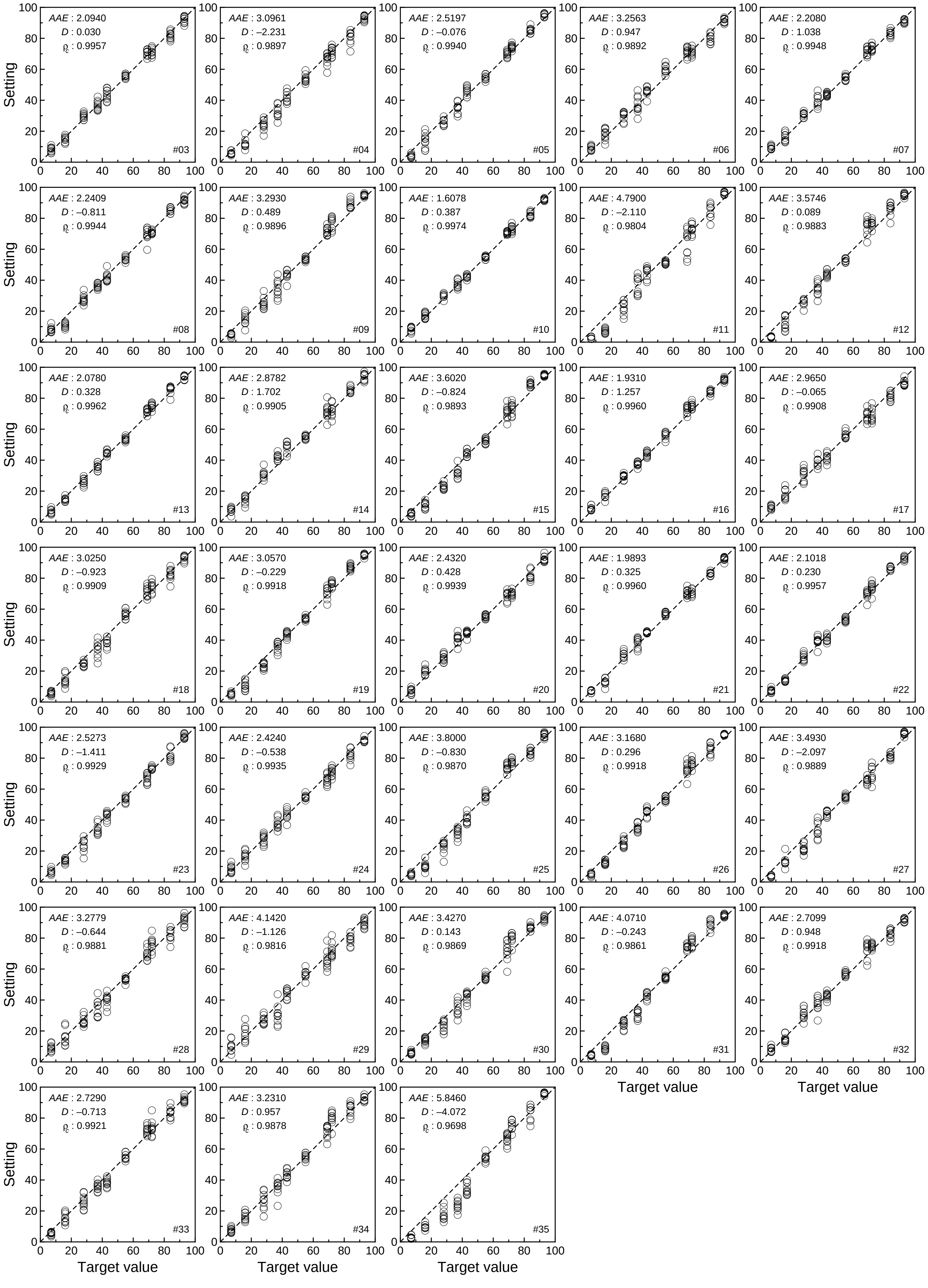

Supplementary Figure S2. Scatter plot of settings against target values for all subjects in the condition without intermediate tick marks. Graphical conventions as in Fig. 1a in the paper.

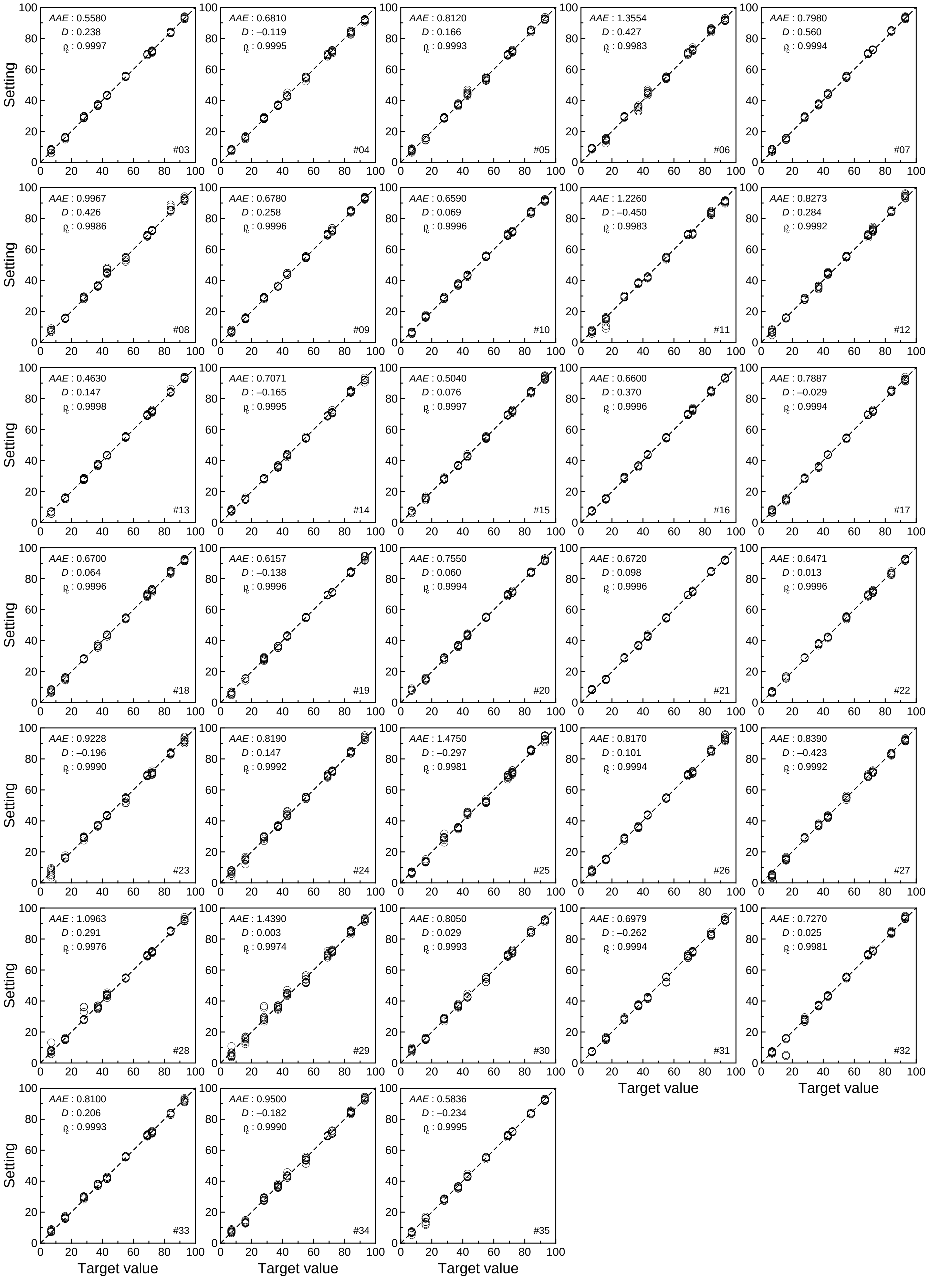

Supplementary Figure S3. Scatter plot of settings against target values for all subjects in the condition with intermediate tick marks. Graphical conventions as in Fig. 1b in the paper.
